# Nitrogen-doped Graphene-Supported Transition-metals Carbide Electrocatalysts for Oxygen Reduction Reaction

**DOI:** 10.1038/srep10389

**Published:** 2015-05-22

**Authors:** Minghua Chen, Jilei Liu, Weijiang Zhou, Jianyi Lin, Zexiang Shen

**Affiliations:** 1 School of Applied Science, Harbin University of Science and Technology, Harbin 150080, P.R. China; 2 Division of Physics and Applied Physics, School of Physical and Mathematical Sciences, Nanyang Technological University, 637371, Singapore; 3 Energy Research Institute @ NTU, Nanyang Technological University, Singapore 639798; 4 School of Mechanical and Aerospace Engineering, Nanyang Technological University, Singapore 637553

## Abstract

A novel and facile two-step strategy has been designed to prepare high performance bi-transition-metals (Fe- and Mo-) carbide supported on nitrogen-doped graphene (FeMo-NG) as electrocatalysts for oxygen reduction reactions (ORR). The as-synthesized FeMo carbide -NG catalysts exhibit excellent electrocatalytic activities for ORR in alkaline solution, with high onset potential (−0.09 V vs. saturated KCl Ag/AgCl), nearly four electron transfer number (nearly 4) and high kinetic-limiting current density (up to 3.5 mA cm^−2^ at −0.8 V vs. Ag/AgCl). Furthermore, FeMo carbide -NG composites show good cycle stability and much better toxicity tolerance durability than the commercial Pt/C catalyst, paving their application in high-performance fuel cell and lithium-air batteries.

Catalysts for the oxygen reduction reaction (ORR) have attracted a great deal of attention because they play an important role in various energy conversion and storage devices, such as fuel cells, water splitting and lithium-air batteries, etc^1^,^2^,^3^,^4^,^5^. Platinum (Pt) based composite materials have been widely used as active ORR catalysts because of their low overpotential and fast kinetics for ORR^6^,^7^. However, the high cost, low durability and poor toxicity tolerance of Pt impede their widespread application in viable commercial progress^8^,^9^. Therefore, it is highly desirable to develop alternative no-precious metal catalysts with high oxygen reduction reaction activity^6^,^10^. Various promising alternative ORR catalysts, including transition metal oxides^11^,^12^,^13^,^14^, transition metal nitrides/carbides^15^,^16^,^17^, transition metal oxynitrides^18^,^19^,^20^, and metal-free heteroatom-doped carbon materials^21^,^22^,^23^, have been explored. Transition-metal carbides have been well studied for catalytic applications because of high electric conductivity, corrosion resistance and “platinum” like behavior for the chemisorptions of hydrogen and oxygen^24^. Tungsten carbides^25^,^26^,^27^, vanadium carbides^28^ and iron carbides^15^ were found to exhibit good ORR activity. Synergistic effects arising from charge transfer have been evidenced when transition metal carbide are used as Pt nanoparticle supports. Although significant progress has been made, the synthesis of nano-sized metal carbides remains a major challenge for the widespread application of transition metal carbide catalysts. Designing new kind of transition metal carbide with enhanced ORR activity in a simple and economical way is therefore meaningful. On the other hand, N-doped graphene (NG) has been reported to be good catalyst as well as good support for ORR because of its large surface area, high electric conductivity, and nitrogen-related active sites^22^,^29^,^30^,^31^,^32^. Therefore, hybrid materials consisting of the transition metal carbides nanoparticles directly grown on the surface of N-doped graphene support would be promising catalyst with improved catalytic performance for ORR.

In this paper, FeMo carbide supported on nitrogen-doped graphene was synthesized successfully via nanoparticle growth on graphene oxide and the subsequent pyrolysis in the presence of urea. The method is simple, facile and easy-processing, which paves the way for the scaled-synthesis of FeMo carbide/NG composite in a low cost way. The FeMo carbide/NG hybrid composites exhibit good electrocatalytic activity, cycling stability and toxic tolerance for oxygen reduction reaction in alkaline solution, which are attributed to the synergic effect between well-dispersed carbide species and N-doped graphene.

## Results

**Figure 1:**
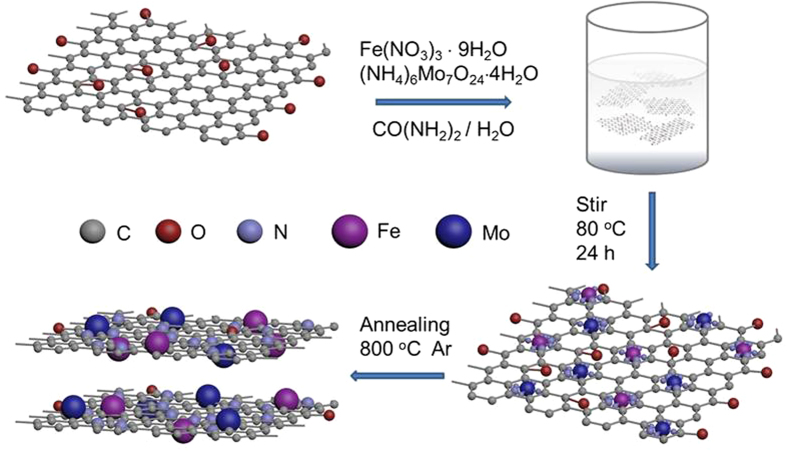
Schematic illustration for preparation of iron- and molybdenum- containing nanoparticles dispersed on nitrogen-doped graphene.

The two-step procedure, described in experimental part for the synthesis of FeMo Carbide/NG, is schematically illustrated in Fig. 1. Urea was added into the reactants solution during the first step to mediate the nucleation of metal species onto the functional group (such as hydroxyl, carboxyl and epoxy groups, etc) of GO. More importantly, urea is a nitrogen source for N-doping of GO (Fig. 1). Subsequent pyrolysis at high temperatures was performed to afford the crystallization of metal carbide nanoparticles, N-doping and reduction of GO, forming designed FeMo Carbide/NG hybrid materials.

**Figure 2:**
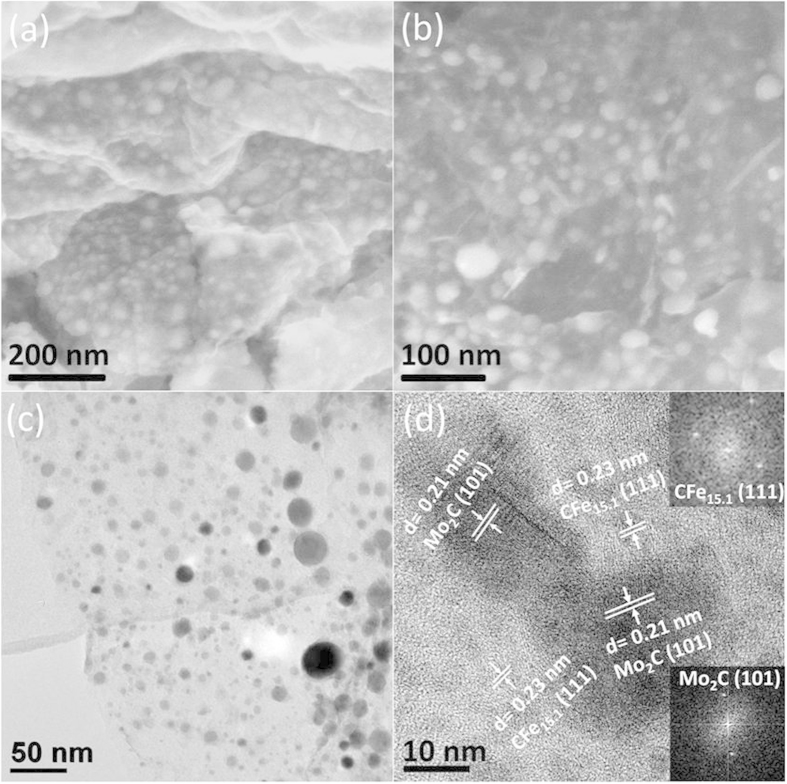
SEM (**a** and **b**), TEM (**c**) and HRTEM images (**d**) of FeMo Carbide/NG-800. The insets in Fig. 2d are SAED patterns of big (bottom) and small (top) nanoparticles, respectively.

The field emission scanning electron microscopy (FESEM) (Fig. 2a,b) and transmission electron microscopy (TEM) (Fig. 2c) of FeMo Carbide/NG-800 which was the sample synthesized at 800 ^o^C with the Fe:Mo molar ratio of 1:1 clearly reveal the uniform distribution of nanopraticles with size between 5 and 20 nm on graphene sheets. In Fig. 2d the bigger particles with lattice space of 0.21 nm corresponding to Mo_2_C (101) and the smaller particles with lattice space of 0.23 nm corresponding to CFe_15.1_ (111) can be observed by HRTEM. This is confirmed by Fast Fourier transform (FFT) and selected area electron diffraction (SAED) patterns (see insets in Fig. 2d). Energy Dispersive Spectrum (EDS) in Fig. S1† can also confirm the presence of Fe, Mo, N and C elements in the sample, with the atomic ratio for Fe:Mo being 1:1, which is consistent well with our designed atomic ratio. Meanwhile, there is high atomic ratio for N in the sample, indicating the successful incorporation of N in the graphene sheets.

**Figure 3:**
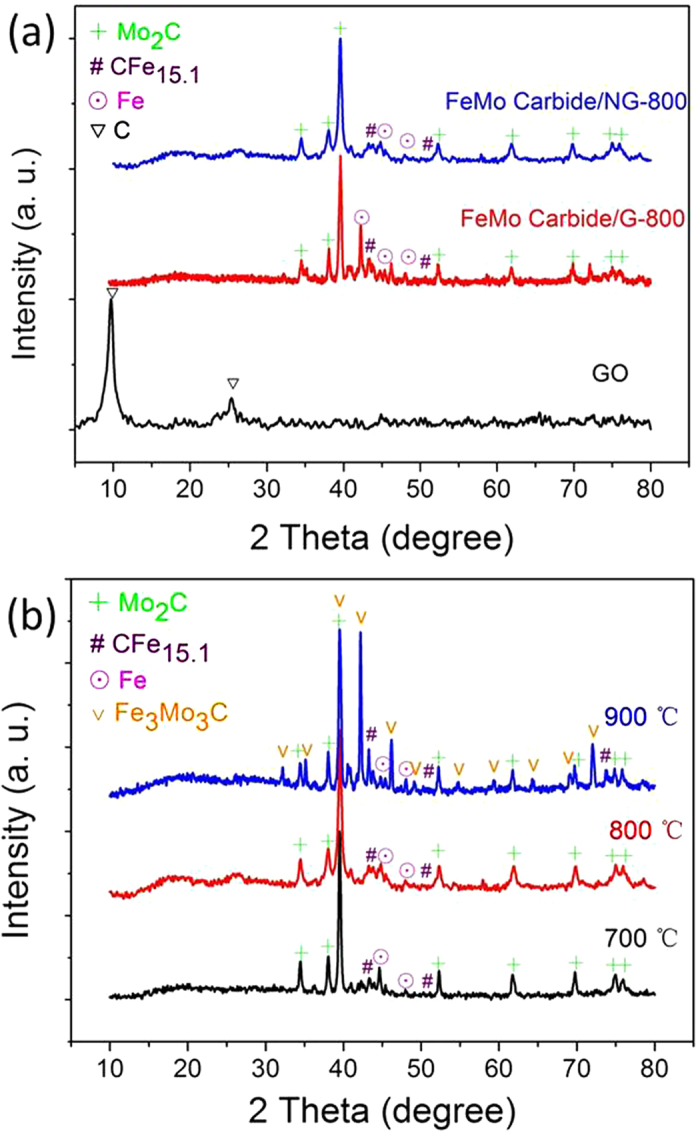
XRD patterns of (**a**) FeMo Carbide/G-800, FeMo Carbide/NG-800 and a dried-down GO. (**b**) FeMo Carbide/NG samples prepared at various annealing temperatures (700, 800 and 900 ^o^C).

XRD patterns of samples GO, FeMo Carbide/G-800 and FeMo Carbide/NG-800 are shown in Fig. 3a. GO displays a dominate diffraction peak at 10.6^o^, corresponding to an interlayer distance of 0.83 nm due to the presence of oxygenated functional groups and the intercalated solvent molecules^34^. For the FeMo-composite materials after mild heat treatment, the GO peak disappeared completely, indicating the reduction of graphite oxide to graphene. The graphite peak disappeared upon high temperature pyrolysis. In Fig. 3a both FeMo Carbide/G-800 and FeMo Carbide/NG-800 show the presence of Mo_2_C (JCPDS card no. 35-0787) as the major species because of the appreance of strong peaks at 34.4 ^o^ (100), 38.0 ^o^ (002), 39.4^o^ (101), 52.1^o^ (102), 61.5^o^ (110), 69.5^o^ (103), 74.6^o^ (112) and 75.5^o^ (201). FeMo Carbide/NG-800 is different from FeMo Carbide/G-800 in N-doping, since urea was added as the N-source for the preparation of FeMo Carbide/NG-800. The Mo_2_C peaks are relatively broadened for FeMo Carbide/NG⊠indicating that Mo_2_C is better dispered on the N-doped graphene support with smaller particle size than that over FeMo Carbide/G, in good consistence to SEM, TEM results (shown in Fig. 2 & S2†) and XRD results (Table S1†). In addition to Mo_2_C, two peaks at 43.3^o^ and 74.1^o^ are observable for both FeMo carbide/NG and FeMo carbide/G, corresponding to CFe_15.1_ (JCPDS card no. 52-0512), indicating the formation of iron carbide from the partial dissolution of carbon atoms into Fe crystal lattices at elevated temperatures^35^. Without the addition of urea some metallic iron phase can be detected in FeMo Carbide/G.

Increasing the annealing temperature of FeMo Carbite/NG from 700 to 900 °C can increase the intensity of characteristic peaks of Mo_2_C sharply, because of the growth of Mo_2_C particles during heating process (see Fig. 3b and Table S1†). Moreover, new diffraction peaks at 32.1^o^, 35.1^o^, 42.1^o^, 46.1^o^, 49.1^o^, 59.2^o^, 69.0^o^ and 71.9^o^, corresponding to Fe_3_Mo_3_C (JCPDS card no. 47-1191), are observed at 900 °C, suggesting the formation of alloy FeMo carbide on NG. As we will discuss later, FeMo Carbide/NG-800, the sample prepared at 800 °C shows the best ORR activity while the weight ratio of Fe/Mo is found to be not as significant as the annealing temperature (Fig. S3†). Therefore our systematic study will focus on FeMo Carbide/NG-800.

Raman spectra of GO, FeMo Carbide/G-800 and FeMo Carbide/NG-800 samples are shown in Fig. S4†. All the samples exhibit two obvious peaks at 1350 and 1590 cm^−1^, corresponding to the graphene D and G bands respectively^33^. The calculated intensity ratios (I_D_/I_G_) for the three samples are 1.40, 1.57, and 1.51, respectively, indicating that the disordering of graphene sheets increases due to the presence of carbides and nitrogen.

**Figure 4:**
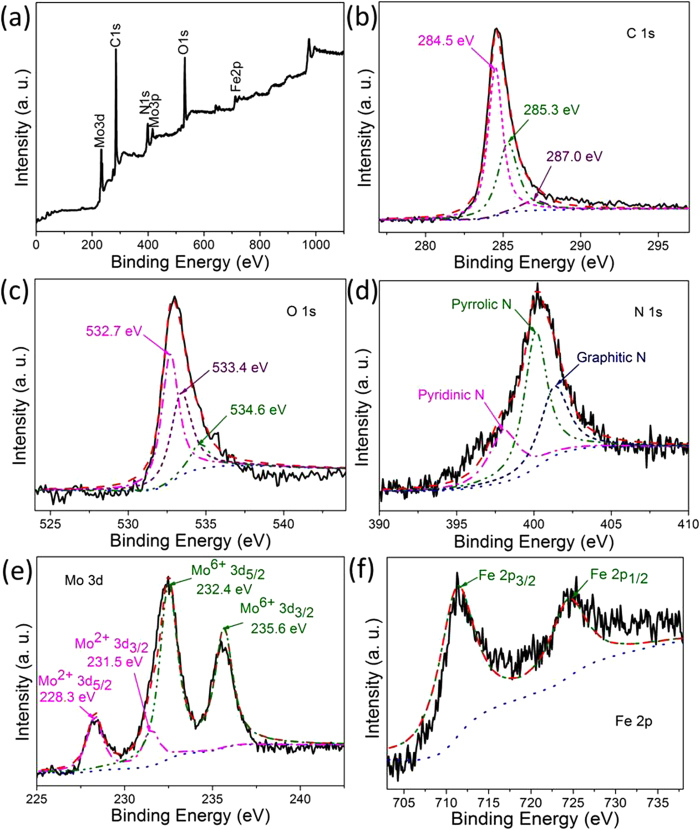
XPS spectra of (**a**) wide scan, (**b**) C 1s , (**c**) O 1s, (d) N 1s, (**e**) Mo 3d and (**f**) Fe 2p of FeMo Carbide/NG-800 catalyst.

XPS spectra in Fig. 4 show the presence of C, O, N, Fe and Mo elements. The C1s peak in Fig. 4b is deconvoluted into three peaks at 284.5, 285.3 and 287.0 eV, which can be assigned to C = C, C-O and -O = C-O respectively^36^. For the O1s spectrum in Fig. 4c, three peaks at binding energy centered at 532.7, 533.4 and 534.6 eV correspond to species C = O/O = C-O, C-OH^37^, and Mo-O respectively. The quantitative XPS analyses of treated samples reveal that the relative amount of oxygen on the catalyst surface decreased slightly with the increase in annealing temperature compared to the XPS of GO (Fig. S5†), suggesting the removal of various functional groups during annealing process. Fig. 4d reveals the presence of pyridinic, pyrrolic and graphitic nitrogen species at 398.1, 400.1 and 401.3 eV, respectively^22^,^29^. The relatively high intensity of the N peak corresponds to a high content (4.7 at%) of doped nitrogen in our NG-related samples (Fig. 4b & Table S2). The Mo3d XPS data (Fig. 4e) exhibits two dominant peaks locating at 232.4 eV and 228.3 eV, which are assigned to Mo^5 + /6 + ^and Mo^2 + ^, respectively, consisting well with previous data reported for molybdenum carbides^38^,^39^. The Mo^2 + ^peak at 228.3 eV is ~4 folds weaker than the high binding energy Mo species in the FeMo Carbide/NG-800 sample (Table S2†), increased relatively with the increase of annealing temperature from 700 to 900 °C, indicating the enhanced formation of Mo_2_C^19^. In Fig. 4f the Fe 2p peaks can be deconvoluted into two peaks at 711.2 and 724.8 eV, corresponding to 2p 3/2 and 2p 1/2 states, respectively^40^.

### Evaluation of elecrocatalytic activity

**Figure 5:**
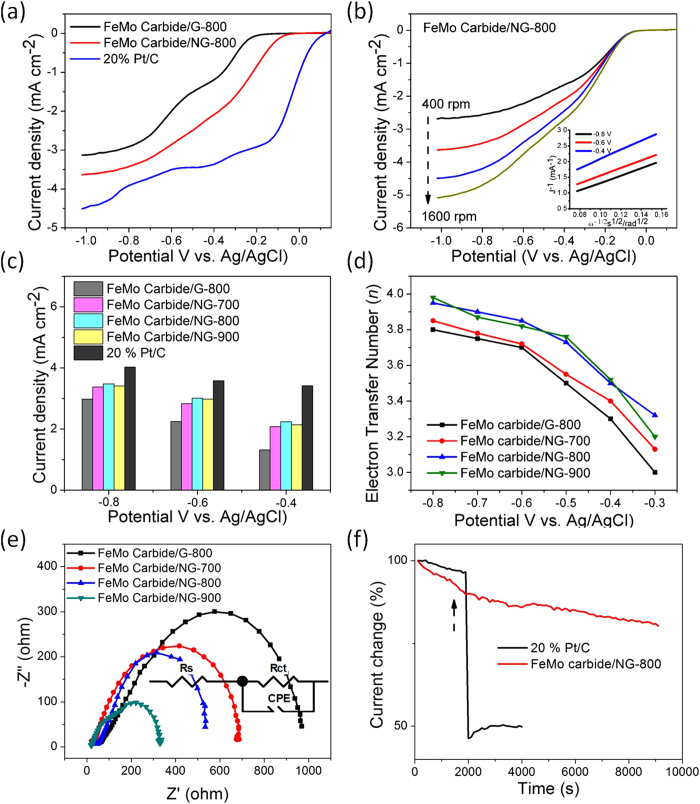
(**a**) RDE polarization curves for ORR performance of FeMo Carbide/G-800, FeMo Carbide/NG-800 and commercial 20% Pt/C in O_2_-saturated 0.1 M KOH with disk rotation rate of 800 rpm. (**b**) RDE curves of the FeMo Carbide/NG-800 at a scan rate of 2 mV s^-1^ with various rotation rates from 400 to 1600 rpm in O_2_-saturated 0.1 M KOH. The inset shows the Tafel plot of the FeMo Carbide/NG-800 derived by the mass-transport correction of the corresponding RDE data. (**c**) The current density J_k_ of 20% Pt/C, FeMo Carbide/G-800, and FeMo Carbide/NG catalysts with various annealing temperatures (700, 800 and 900 ^o^C) at two different potentials, -0.8, -0.6 and -0.4 V respectively. (**d**) The dependence of electron transfer number (n) on the potential for FeMo Carbide/G-800 and FeMo Carbide/NG catalysts with different annealing temperatures (700, 800 and 900 ^o^C) and corresponding electrochemical impedance spectroscopy measured at -0.30 V (e). The inset of (**e**) shows an equivalent electrical circuit consisting of the electrolyte resistance (R_s_), interface charge transfer resistance (R_ct_) and constant phase element (CPE). (**f**) Current (i)–time (t) chronoamperometric responses obtained for both FeMo Carbide/NG-800 (black line) and 20% Pt/C catalysts (red line) at -0.55 V in O_2_-saturated 0.1 M KOH solution to methanol. The arrow indicates the addition of 2% (weight ratio) methanol into the O_2_-saturated electrochemical cell.

The oxygen reduction reaction (ORR) activity of various graphene-supported FeMo carbide materials was evaluated by cyclic voltammetry (CV) (Fig. S6) and rotating disk electrode (RDE) measurements (Fig. 5)^41^, respectively. Compared with featureless CV curves in N_2_-saturated 0.1 M KOH solution (black line), well-defined oxygen reduction peaks were observed for all electrodes in the O_2_-saturated KOH solution (red line), revealing the ORR catalytic activity of the graphene-supported FeMo carbide materials. It was noted that the cathodic current of CV curves for all graphene-supported FeMo carbide electrodes display similar reduction peak between −0.5 and −0.3 V (vs. Ag/AgCl), which is attributed to the electrocatalytic oxygen reduction on the electrode^23^,^28^,^30^,^42^. However, the corresponding onset potential, half-wave potential and diffusion-limited currents are different for various electrodes, depending on the catalyst materials. As seen from the results in Fig. S6 and Table S4†, the onset (E1) and half-wave potentials (E2) are the E1 = −0.20 V and E2 = −0.51 V for N-free FeMo Carbide/G-800 (Fig. S6a &Table S4), respectively, lower than those of N-doped graphene supported FeMo carbides, i.e. E1 = −0.14 to −0.09 V and E2 = −0.37 to −0.33 V for FeMo Carbides/NG (Figs. S6 b-f &Table S4). The up-shift of on-set and half-wave potentials clearly indicates that N-doping can enhance catalyst performance for ORR. Moreover, the reduction peaks between −0.5 and −0.3 V become more clear and well-defined with the increasing annealing temperature from 700 to 900 degree, which is attributed to the increased N-doping level (4.2, 4.7 and 4.9% respectively in Table S2†) and thus the enhanced electric conductivity of the catalysts (see impedance data in Fig. 5e). The abovementioned results were further verified via linear sweep voltammery (LSV) measurements on a rotating disk electrode (RDE) for deferent graphene-supported FeMo carbide materials, along with the commercial Pt/C electrode, at a scan rate of 2 mV/s in O_2_-saturated 0.1 M KOH solution. The polarization curves of graphene-supported FeMo carbide electrodes in Fig. 5a exhibits two-step process, indicating a mixture of two-electron and four-electron reduction processes. The onset potential and half-wave potential for FeMo Carbide/G-800 are E1 = −0.20 V and E2 = −0.51 V, remarkably lower than those of FeMo Carbide/NG-800 in Fig. 5a (i.e. E1 = −0.09 V and E2 = −0.33 V). The current density for FeMo Carbide/G-800 is 1.3 mA/cm^2^ at −0.40 V, 2.3 mA/cm^2^ at −0.6 V, and 3.0 mA/cm^2^ at −0.8 V, respectively, much lower than 2.3 mA/cm^2^ (at −0.40 V), 3.0 mA/cm^2^ (at −0.60 V) and 3.5 mA/cm^2^ (at −0.80 V) for FeMo Carbide/NG-800 (see Fig. 5c & Table S4†). The enhanced ORR current density on FeMo Carbide/NG-800 appears to be attributed to the better dispersion of metal carbides and hence more active sites on NG-800 (vs. G-800). Nevertheless both FeMo Carbides on G-800 and NG-800 catalysts in Fig. 5a are still not comparable to commercial Pt/C catalysts. The polarization profile of Pt/C in Fig. 5a shows much better ORR performance with high onset potential (E1 = 0.06 V), high half-wave potential (E2 = −0.08 V) and high diffusion-limited current (4.0 mA/cm^2^ at −0.8 V).

In Fig. 5b RDE profiles of FeMo Carbide/NG-800 at 2 mV s^−1^ in O_2_-saturated 0.1 M KOH increases the current density with increasing rotating rates from 400 to 1600 rpm, indicating kinetic-limiting behavior of ORR. The corresponding Koutecky-Levich plots (inset in Fig. 5b) at different potentials exhibit good linearity, suggesting the first-order reaction kinetics towards the concentration of dissolved oxygen in the electrolyte solution^29^,^43^. Note that the slopes of Koutecky-Levich plots are not consistent, indicating that the electron transfer numbers for ORR at different potentials are different. The electron transfer number (*n*) derived from the Koutecky-Levich plots at potential between -0.8 and -0.3 V is calculated and plotted in Fig. 5d. At potential of −0.53 V, 3.5 electron transfer number is obtained, suggesting a mixture of two-electron and four-electron reduction processes, in a good agreement with CV and LSV curves. The electron transfer number then increases with increasing negative potential, reaching 3.9 at −0.73 V, indicating four-electron reduction dominated in the high negative potential range^21^. In the whole potential range the n number is higher for FeMo Carbide/NG-800 than FeMo Carbide/G-800, suggesting that N-doping can promote the electron transfer from the catalysts to O_2_. Moreover, FeMo Carbide/NG-800 exhibits excellent cycling stability with slightly decrease in limiting current density (from 3.5 mA/cm^2^ to 3.2 mA/cm^2^ at −0.8 V) and nearly no change in on-set potential (Fig. S7†) after 1,000 cycles, corroborating its further commercial applications.

The enhanced electrocatalytic activity of FeMo Carbide/NG-800 vs. G-800 can be attributed to three reasons: (i) The incorporation of nitrogen into graphene in terms of graphitic N, pyridinic N and pyrrolic-like N (Table S2† & S3†) enhances the bonding ability of the carbon atoms adjacent to the N atoms, favouring the absorption and activation of dioxygen to OOH, H_2_O_2_ and OH, which improves its ORR activity^22^,^30^,^44^; (ii) The strong interaction between Mo oxyanions and N-atoms or oxygen-containing functional groups could prevent Mo_2_C or CFe_15.1_ nanoparticles from agglomeration during annealing process, which promotes the well-dispersion of metal species on the N-doping graphene support and thus provides more metal-based active sites for ORR^45^; (iii) The N-doping improves the electric conductivity of composites materials, providing a highly conductive pathway for electrons transfer. The electrochemical impedance spectra of the catalysts were measured at −0.30 V and presented in Fig. 5e. A kinetics-based model consisting of solution resistance (R_s_), faradaic resistance (R_ct_) and constant phase element (CPE) was adopted to fit the impedance spectra. Indeed, a sharp decrease in faradaic resistance for FeMo Carbide/NG-800 (659 Ω) could be observed compared to that of FeMo Carbide/G-800 (~1126 Ω).

In Figs. 5 and S6 the ORR activities of NG-supported FeMo Carbide composites that were prepared under various temperatures (700–900 °C) with fixed 1:1 Mo/Fe atomic ratio are compared. The sample treated at 800 °C (FeMo Carbide/NG-800) was found to exhibit the best activity in terms of onset potential, half-wave potential and limiting current density. For the sample treated at 700 °C (FeMo Carbide/NG-700) (Fig. S8†&Table S4), the onset potential is located at around −0.14 V, which is about 50 mV lower than that of FeMo Carbide/NG-800, but still better than that of FeMo Carbide composite without N-doping (with onset potential at −0.20 V, see Table S4†). Furthermore, an improvement in onset potential has been observed for FeMo Carbide/NG-900 (Fig. S9†&Table S4, onset potential of −0.12 V), which is attributed to the improvement in catalytic performance resulting from remove of oxygen function groups and increase in nitrogen content. This could be confirmed by XPS results that the oxygen content reduced from 17.0 atom % for FeMo carbide/NG-700 to 15.0 atom % for FeMo carbide/NG-900 while the N content increased from 4.2 atom % to 4.9 atom %. Correspondingly the faradic resistance decreased from 714 Ω for FeMo Carbide/NG-700 to 351 Ω for FeMo Carbide/NG-900 while higher limiting current density is observable in Fig. S8, S9 and Table S4 for the NG-900 catalyst than the NG-700.

In addition to the pyrolysis temperature, the Fe/Mo ratio also impacts ORR activity of hybrid materials. FeMo (1:3) Carbide/NG-800 (Fig. S10 & Table S4†) and FeMo (3:1) Carbide/NG-800 (Fig. S11† & Table S4†) both displayed moderate activity with onset potential locate at −0.11 V and −0.14 V, respectively, lower than that (−0.09 V) of FeMo Carbide/NG-800. However, FeMo catalysts are both superior over single-component carbide, i.e. Fe carbide/NG-800 (−0.19 V) and Mo carbide/NG-800 (−0.17 V) (Table S4†), suggesting that the co-existence of Fe and Mo carbides enhances the ORR activity. It is still not very clear in what way the Mo/Fe ratio affect the onset potential at the moment. However, an increase in limiting current density at −0.8 V has been observed with the decrease of Fe/Mo ratio in the order: FeMo (1:3) Carbide/NG-800 (3.6 mA/cm^2^) > FeMo (3:1) Carbide/NG-800 (3.2 mA/cm^2^) > Mo Carbide/NG-800 (2.7 mA/cm^2^) > Fe Carbide/NG-800 (2.5 mA/cm^2^), indicating that molybedum contribute more than iron to ORR activity. Similar trend was also observed in faradic resistance changes (Fig. S12†). 342 Ω was observed for sample for FeMo (1:3) carbide/NG-800, which is much smaller than that of for FeMo (3:1) carbide/NG-800. These founding here would be helpful for catalyst design in future.

FeMo Carbide/NG-800 was further subjected to testing of methanol crossover in order to confirm its feasibility for ORR applications (Fig. 5f). At a constant voltage of −0.55 V (vs. Ag/AgCl), the response current of the hybrid catalyst decrease by only 18% over 90,000s, while the commercial 20% Pt/C catalyst exhibited a sharp current decrease upon the addition of 2% wt methanol into electrolyte, suggesting the good stability and toxicity tolerance for ORR in alkaline solutions. This is partly because of the much lower ORR potential than that required for oxidization of methanol on FeMo carbide/NG-800 electrodes^10^ and the high catalytic activity of the Pt catalyst for methanol oxidation^46^. Since methanol molecules could be adsorbed more easily on the surface of Pt catalyst, and thus the adsorption of oxygen molecules at the active sites would be interrupted, resulting in a significant activity loss in the Pt catalyst-based DMFC^47^. Although the intrinsic catalytic activity of FeMo carbide/NG-800 is still poorer than that of Pt/C, the elimination of performance losses associates with methanol crossover make it a promising candidate for oxygen reduction reaction catalyst.

## Discussion

We demonstrated that nanosized crystalline FeMo Carbides such as Mo_2_C, Fe_15.1_C and Fe_3_Mo_3_C could be easily synthesized by employing graphene as both support and carbon source, through a facile two-step procedure combining nanoparticle growth on graphene sheets and nitrogen-doping of graphene during pyrolysis process. XRD, TEM and XPS results reveal that the addition of urea can not only provides N sources, but also prevents nanosized Mo_2_C from aggregation during annealing process because of the strong interaction between Mo oxyanions and N-atoms or oxygen-containing functional groups. The FeMo carbide/NG hybrid composites obtained exhibit good electrocatalytic activity, cycling stability and toxic tolerance for oxygen reduction reaction in alkaline solution, which are attributed to the synergic effect between well-dispersed carbide species and N-doped graphene. N-doping was found to play an important role in enhanced ORR performance by improving the electron transfer and increasing the density of active sites of the composites materials. Additionally, it was found that the electrocatalytic performance is strongly dependent on pyrolysis temperature and the Fe/Mo weight ratio. Particularly, corresponding limiting current density at -0.8 V increase with the decrease of Fe/Mo weight ratio in the order: FeMo (1:3) Carbide/NG-800 (3.6 mA/cm^2^) > FeMo (3:1) Carbide/NG-800 (3.2 mA/cm^2^) > Mo Carbide/NG-800 (2.7 mA/cm^2^) > Fe Carbide/NG-800 (2.5 mA/cm^2^), indicating that molybedum contribute more than iron to ORR activity. These results presented here would be very helpful for guiding catalyst design with optimized performance in future.

## Methods

### Preparation of Graphene Oxide (GO)

GO was prepared by a modified Hummers method from natural flake graphite powder^22^. Briefly, 5 g of graphite was mixed with 300 ml of concentrated H_2_SO_4_ in an ice bath for 1 h. 10 g of KMnO_4_ was then added into the above solution slowly under continuous stirring. The mixture was then kept at 90 °C for 4h, washed with 5 wt% HCl and DI water, and freeze-dried for further use.

### Preparation of FeMo Carbide/NG

0.2 g of Fe(NO_3_)_3_ · 9H_2_O (Merck AR, > 99.0%) and 0.088 g of (NH_4_)_6_Mo_7_O_24_ · 4H_2_O (AR, >99.0%) were dispersed in DI water (50 ml) and pretreated with ultrasonication for 0.5 h. After addition of 0.3 g of urea (AR, >99.0%), the mixture was stirred for another 1 h. Subsequently, the aqueous solution of GO (100 mg) dispersed in DI water (50 ml) after pretreated with ultrasonication for 0.5 h was added slowly into the above solution. After 12 hours constant stirring at 80 °C, the resultant solution was freeze-dried and collected for pyrolysis in an Ar/H_2_ stream at flow rate of 100 sccm for 2 h to form FeMo Carbide/Nitrogen-doped Graphene hybrid sample (denoted as FeMo Carbide/NG-n, n is the annealing temperature).

Samples with various Fe/Mo atomic ratios (such as 4:0, 0:4, 1:3, 1:1 and 3:1) were also prepared under identical conditions at 800 °C. The corresponding samples were denoted as Fe Carbide/NG-800, Mo carbide/NG-800, FeMo (1:3) Carbide /NG-800, FeMo Carbide/NG-800 and FeMo (3:1) Carbide/NG-800, respectively.

### Characterization

Material Characterization: The sample morphologies were studied by field-emission scanning electron microscope (FE-SEM JEOL JSM-6700F; JEOL, Tokyo, Japan). The Raman spectra were recorded on a WITEC-CRM200 Raman system (WITEC, Germany), using 532 nm laser (2.33 eV) as the excitation source. The crystal structure of the samples was examined by a Bruker D8 ADVANCE XRD. Average crystallite size was calculated according to the Scherrer’s equation, *D = k*λ/(Bcosθ), where *k* is a constant corresponding to the shape of the polycrystals (here chosen as 0.9), B is the full-width at half maximum (FWHM) of the respective diffraction peak, θ is the Bragg angle. The surface chemical composition of the samples was determined by x-ray photoelectron spectroscopy (XPS) on a VG ESCALAB 250 spectrometer (Thermo Electron, UK), using Al Kα X-ray source (1486 eV).

Electrochemical Characterization: Oxygen reduction reaction (ORR) measurements were carried out at room temperature on a rotating-disk three-electrode system (Eco Chemie, Netherland) in an O_2_-saturated 0.1 M KOH aqueous electrolyte using Ag/AgCl reference electrode^29^. The electrolyte was bubbled with purified oxygen flow at around 140 sccm for about 30 min before every experiment. The oxygen flow was then kept constant at 140 sccm throughout the whole measurement.

The working electrode was prepared using the method described in literature^33^. Briefly, 10 mg of the catalyst was dispersed into 1 mL of 2-propanol containing a Nafion solution (5 wt%, DuPont) with the aid of ultrasonication. 10 μL of the catalyst ink was then coated on the glassy carbon disc electrode (5 mm in diameter, Eco Chemie, Netherlands) and dried at 60 °C. The mass loading of active materials is 100 μg.

The ORR stability test of both FeMo carbide/NG and 20% Pt/C were performed in 0.1 M KOH between −1.0 to 0.2 V (vs. saturated KCl Ag/AgCl) with the scan rate of 2 mV/s for 1,000 cycles. The Koutecky–Levich equation was used to calculate the number of electron transferred (n) (see Supporting Information).

## Author Contributions

M.H.C. and J.L.L. contributed equally to this work. J.L.L. and J.Y.L. conceived and designed the experiments. M.H.C., J.L.L. and W.J.Z. synthesized the catalysts, conducted materials characterizations (SEM, EDS, XPS, HRTEM, XRD and Raman) and electrochemical measurements. J.L.L., M.H.C. and Z.X.S. wrote the manuscript. All authors reviewed the manuscript.

## Additional Information

**How to cite this article**: Chen, M. *et al.* Nitrogen-doped Graphene-Supported Transition-metals Carbide Electrocatalysts for Oxygen Reduction Reaction. *Sci. Rep.* **5**, 10389; doi: 10.1038/srep10389 (2015).

## Supplementary Material

Supporting Information

## References

[b1] Guo S. J., Zhang S. & Sun S. S. Tuning Nanoparticle Catalysis for the Oxygen Reduction Reaction. Angew. Chem. Int. Ed. 52, 8526–8544 (2013). 10.1002/anie.201207186 23775769

[b2] Jaouen F. *et al.* Recent advances in non-precious metal catalysis for oxygen-reduction reaction in polymer electrolyte fuel cells. Energy Environ. Sci. 4, 114–130 (2011).

[b3] Zheng Y., Jiao Y., Jaroniec M., Jin Y. G. & Qiao S. Z. Nanostructured Metal-Free Electrochemical Catalysts for Highly Efficient Oxygen Reduction. Small 8, 3550–3566 (2012). 22893586 10.1002/smll.201200861

[b4] Liu J. L. *et al.* Carbon Nanotube-Based Materials for Fuel Cell Applications. Aust. J. Chem. 65, 1213–1222 (2012).

[b5] Zhu C. Z. & Dong S. J. Recent progress in graphene-based nanomaterials as advanced electrocatalysts towards oxygen reduction reaction. Nanoscale 5, 1753–1767 (2013). 23364753 10.1039/c2nr33839d

[b6] Wu G., More K. L., Johnston C. M. & Zelenay P. High-Performance Electrocatalysts for Oxygen Reduction Derived from Polyaniline, Iron, and Cobalt. Science 332, 443–447 (2011). 21512028 10.1126/science.1200832

[b7] Zhang J., Sasaki K., Sutter E. & Adzic R. R. Stabilization of platinum oxygen-reduction electrocatalysts using gold clusters. Science 315, 220–222 (2007). 17218522 10.1126/science.1134569

[b8] Schmittinger W. & Vahidi A. A review of the main parameters influencing long-term performance and durability of PEM fuel cells. J. Power Sources 180, 1–14 (2008).

[b9] Yu X. W. & Ye S. Y. Recent advances in activity and durability enhancement of Pt/C catalytic cathode in PEMFC: Part II: Degradation mechanism and durability enhancement of carbon supported platinum catalyst. J. Power Sources 172, 145–154 (2007).

[b10] Gong K. P., Du F., Xia Z. H., Durstock M. & Dai L. M. Nitrogen-Doped Carbon Nanotube Arrays with High Electrocatalytic Activity for Oxygen Reduction. Science 323, 760–764 (2009). 19197058 10.1126/science.1168049

[b11] Suntivich J. *et al.* Design principles for oxygen-reduction activity on perovskite oxide catalysts for fuel cells and metal-air batteries. Nat. Chem. 3, 546–550 (2011). 21697876 10.1038/nchem.1069

[b12] Guo S. J., Zhang S., Wu L. H. & Sun S. S. Co/CoO Nanoparticles Assembled on Graphene for Electrochemical Reduction of Oxygen. Angew. Chem. Int. Ed. 51, 11770–11773 (2012). 10.1002/anie.201206152 23073995

[b13] Liang Y. Y. *et al.* Covalent Hybrid of Spinel Manganese-Cobalt Oxide and Graphene as Advanced Oxygen Reduction Electrocatalysts. J. Am. Chem. Soc. 134, 3517–3523 (2012). 22280461 10.1021/ja210924t

[b14] Zhang G. Q., Xia B. Y., Wang X. & Lou X. W. Strongly Coupled NiCo2O4-rGO Hybrid Nanosheets as a Methanol-Tolerant Electrocatalyst for the Oxygen Reduction Reaction. Adv. Mater. 26, 2408–2412 (2014). 24338831 10.1002/adma.201304683

[b15] Kramm U. I. *et al.* Effect of iron-carbide formation on the number of active sites in Fe-N-C catalysts for the oxygen reduction reaction in acidic media. J. Mater. Chem. A 2, 2663–2670 (2014).

[b16] Tsai C. W. *et al.* Carbon incorporated FeN/C electrocatalyst for oxygen reduction enhancement in direct methanol fuel cells: X-ray absorption approach to local structures. Electrochim. Acta 56, 8734–8738 (2011).

[b17] Xia D. G. *et al.* Methanol-tolerant MoN electrocatalyst synthesized through heat treatment of molybdenum tetraphenylporphyrin for four-electron oxygen reduction reaction. J. Power Sources 177, 296–302 (2008).

[b18] Chisaka M., Suzuki Y., Iijima T. & Sakurai Y. Effect of Synthesis Route on Oxygen Reduction Reaction Activity of Carbon-Supported Hafnium Oxynitride in Acid Media. J. Phys. Chem. C 115, 20610–20617 (2011).

[b19] Cao B. F. *et al.* Cobalt Molybdenum Oxynitrides: Synthesis, Structural Characterization, and Catalytic Activity for the Oxygen Reduction Reaction. Angew. Chem. Int. Ed. 52, 10753–10757 (2013). 10.1002/anie.201303197 24038934

[b20] Ishihara A. *et al.* Tantalum oxynitride for a novel cathode of PEFC. Electrochem. Solid. St. Lett . 8, A201–A203 (2005).

[b21] Liu R. L., Wu D. Q., Feng X. L. & Mullen K. Nitrogen-Doped Ordered Mesoporous Graphitic Arrays with High Electrocatalytic Activity for Oxygen Reduction. Angew. Chem. Int. Edit. 49, 2565–2569 (2010). 10.1002/anie.200907289 20217877

[b22] Lai L. F. *et al.* Exploration of the active center structure of nitrogen-doped graphene-based catalysts for oxygen reduction reaction. Energy Environ. Sci. 5, 7936–7942 (2012).

[b23] Jeon I. Y. *et al.* Facile, scalable synthesis of edge-halogenated graphene nanoplatelets as efficient metal-free eletrocatalysts for oxygen reduction reaction. Sci. Rep. 3, 1810 (2013). 23736800 10.1038/srep01810 PMC3672906

[b24] Levy R. B. & Boudart M. Platinum like behavior of tungsten carbide in surface catalysis. Science 181, 547–549 (1973). 17777803 10.1126/science.181.4099.547

[b25] Yan Y. *et al.* Nano-tungsten carbide decorated graphene as co-catalysts for enhanced hydrogen evolution on molybdenum disulfide. Chem. Commun. 49, 4884–4886 (2013). 10.1039/c3cc41031e 23535746

[b26] Meng H. & Shen P. K. Novel Pt-free catalyst for oxygen electroreduction. Electrochem. Commun. 8, 588–594 (2006).

[b27] Yan Z. X. *et al.* A facile route to carbide-based electrocatalytic nanocomposites. J. Mater. Chem. 22, 5072–5079 (2012).

[b28] Huang T. Z. *et al.* Nitrogen-doped graphene-vanadium carbide hybrids as a high-performance oxygen reduction reaction electrocatalyst support in alkaline media. J. Mater. Chem. A 1, 13404–13410 (2013).

[b29] Liu J. L. *et al.* Improved synthesis of graphene flakes from the multiple electrochemical exfoliation of graphite rod. Nano Energy 2, 377–386 (2013).

[b30] Feng L. Y. *et al.* Enhancing Electrocatalytic Oxygen Reduction on Nitrogen-Doped Graphene by Active Sites Implantation. Sci. Rep . 3, 3306 (2013). 24264379 10.1038/srep03306 PMC3837309

[b31] Zhang C. Z., Hao R., Yin H., Liu F. & Hou Y. L. Iron phthalocyanine and nitrogen-doped graphene composite as a novel non-precious catalyst for the oxygen reduction reaction. Nanoscale 4, 7326–7329 (2012). 23086132 10.1039/c2nr32612d

[b32] Park M., Lee T. & Kim B. S. Covalent functionalization based heteroatom doped graphene nanosheet as a metal-free electrocatalyst for oxygen reduction reaction. Nanoscale 5, 12255–12260 (2013). 24146109 10.1039/c3nr03581f

[b33] Marcano D. C. *et al.* Improved Synthesis of Graphene Oxide. Acs Nano 4, 4806–4814 (2010). 20731455 10.1021/nn1006368

[b34] Prakash. R. *et al.* A ferrocene-based carbon-iron lithium fluoride nanocomposite as a stable electrode material in lithium batteries. J. Mater. Chem. 20, 1871–1876 (2010).

[b35] Liu J. L. *et al.* A green approach to the synthesis of high-quality graphene oxide flakes via electrochemical exfoliation of pencil core. RSC Advances 3, 11745–11750 (2013).

[b36] Poh C. K. *et al.* Nanostructured trimetallic Pt/FeRuC, Pt/NiRuC, and Pt/CoRuC catalysts for methanol electrooxidation. J. Mater. Chem. 22, 13643–13652 (2012).

[b37] Yang D. X. *et al.* Chemical analysis of graphene oxide films after heat and chemical treatments by X-ray photoelectron and Micro-Raman spectroscopy. Carbon 47, 145–152 (2009).

[b38] Xiang M. L. *et al.* XPS study of potassium-promoted molybdenum carbides for mixed alcohols synthesis via CO hydrogenation. J. Nat. Gas. Chem. 19, 151–155 (2010).

[b39] Hada K., Nagai M. & Omi S. Characterization and HDS activity of cobalt molybdenum nitrides. J. Phys.Chem. B 105, 4084–4093 (2001).

[b40] Fujii T., Groot F. M. F. & Sawatzky G. A. In situ XPS analysis of various iron oxide films grown by NO2-assisted molecular-beam epitaxy. Phys. Rev. B 59, 3195–3202 (1999).

[b41] Snyder J., Fujita T., Chen M. W. & Erlebacher J. Oxygen reduction in nanoporous metal–ionic liquid composite electrocatalysts. Nat. Mater. 9, 904–907 (2010). 20953182 10.1038/nmat2878

[b42] Peng H. L. *et al.* High Performance Fe- and N- Doped Carbon Catalyst with Graphene Structure for Oxygen Reduction. Sci. Rep. 3, 1765 (2013).

[b43] Liang Y. Y. *et al.* Co3O4 nanocrystals on graphene as a synergistic catalyst for oxygen reduction reaction. Nat. Mater. 10, 780–786 (2011). 21822263 10.1038/nmat3087

[b44] Jin H., Zhang H. M., Zhong H. X. & Zhang J. N. Nitrogen-doped carbon xerogel: A novel carbon-based electrocatalyst for oxygen reduction reaction in proton exchange membrane (PEM) fuel cells. Energy Environ. Sci. 4, 3389–3394 (2011).

[b45] Li X. Y., Ma D., Chen L. M. & Bao X. H. Fabrication of molybdenum carbide catalysts over multi-walled carbon nanotubes by carbothermal hydrogen reduction. Catal. Lett. 116, 63–69 (2007).

[b46] Gupta S. *et al.* Methanol-tolerant electrocatalysts for oxygen reduction in a polymer electrolyte membrane fuel cell. J. Appl. Electrochem. 28, 673–682 (1998).

[b47] Reeve R. W., Christensen P. A., Hamnett A., Haydock S. A. & Roy S. C. Methanol Tolerant Oxygen Reduction Catalysts Based on Transition Metal Sulfides. J. Electrochem. Soc. 145, 3463–3471 (1998).

