# Supplementary material for: Nitrogen-doped Graphene-Supported Transition-metals Carbide Electrocatalysts for Oxygen Reduction Reaction
Source: Sci Rep. 2015 May 22;5:10389. doi: 10.1038/srep10389 (PMC4441168; doi:10.1038/srep10389)
Supplement: Supporting Information [file srep10389-s1.doc]

Supporting information

**Nitrogen-doped Graphene-Supported Transition-metals Carbide Electrocatalysts for Oxygen Reduction Reactions**

Minghua Chen ^a,b†^, Jilei Liu ^b, c†^, Weijiang Zhou ^d^, Jianyi Lin ^c^*^*^*, Zexiang Shen ^b*^

^a^ School of Applied Science, Harbin University of Science and Technology, Harbin 150080, P.R. China

^b^ Division of Physics and Applied Physics, School of Physical and Mathematical Sciences, Nanyang Technological University, 637371, Singapore

^c^ Energy Research Institute @ NTU, Nanyang Technological University, Singapore 639798

^d^ School of Mechanical and Aerospace Engineering, Nanyang Technological University, Singapore 637553

^†^ These two authors contributed equally to this work.

*^*^*Corresponding Authors. Email: [LiJY@ntu.edu.sg](mailto:LiJY@ntu.edu.sg) (J. L. Liu) and [zexiang@ntu.edu.sg](mailto:zexiang@ntu.edu.sg) (Z. S. Shen).

**1 Electron transfer number calculation**

The electron transfer number can be calculated from linear voltammograms as

(1)

where j_k_ is the kinetic current density, =Bω^1/2^ is the limiting diﬀusion current density, B is the Levich constant, ω is the rotation rate of electrode, n is the number of exchanged electrons per oxygen molecule, and 0.2 is the coefficient used when ω is expressed in rpm. A plot of 1/j against ω^-1/2^ (Koutecky-Levich plot) can be used to determine the value of n (electron transfer number). The number of electrons transferred per oxygen molecule can be calculated from Levich constant via the following equation

B=0.62⋅n⋅F⋅D⋅^2/3^⋅ν^-1/6^⋅C (2)

where F, the Faradic constant, equals 96486 C mol^-1^, D is the diffusion coefficient O_2_ in the electrolyte (1.93 × 10^-5^ cm^2^ s^-1^), ν is the kinematic viscosity of the sulfuric acid (0.01 cm^2^ s^-1^), C is the concentration of dissolved oxygen (1.26 × 10^-6^ mol cm^-3^).

**2 Figures and images:**


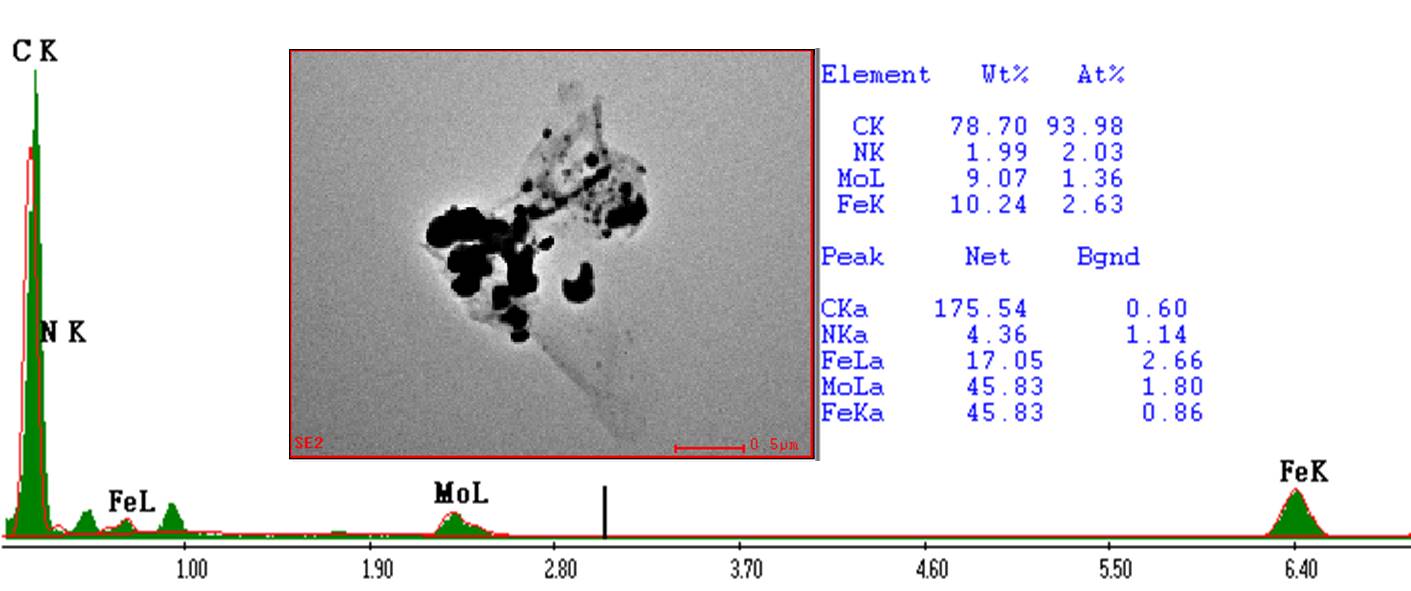


**Fig. S1** EDS spectrum of FeMo Carbide/NG-800 catalyst.


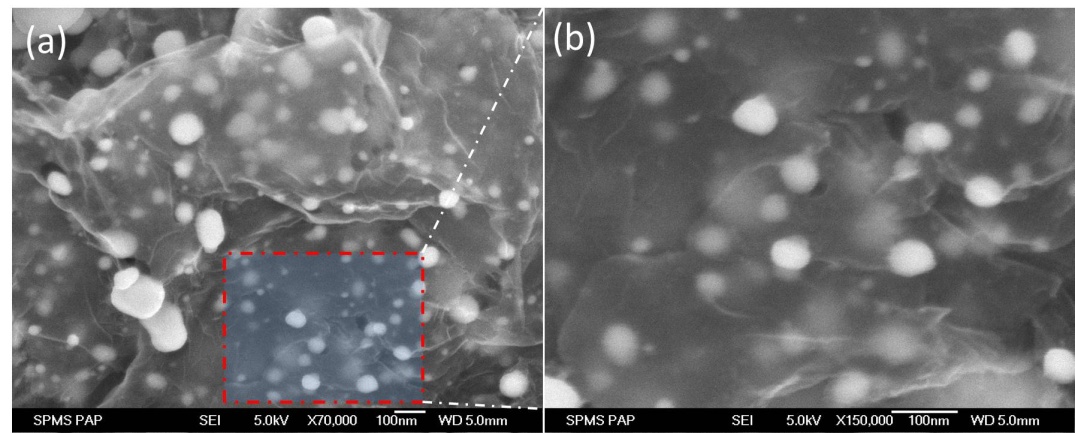


**Fig. S2** SEM images (a) Low-magnification and (a) High-magnification of FeMo Carbide/G-800 catalyst.


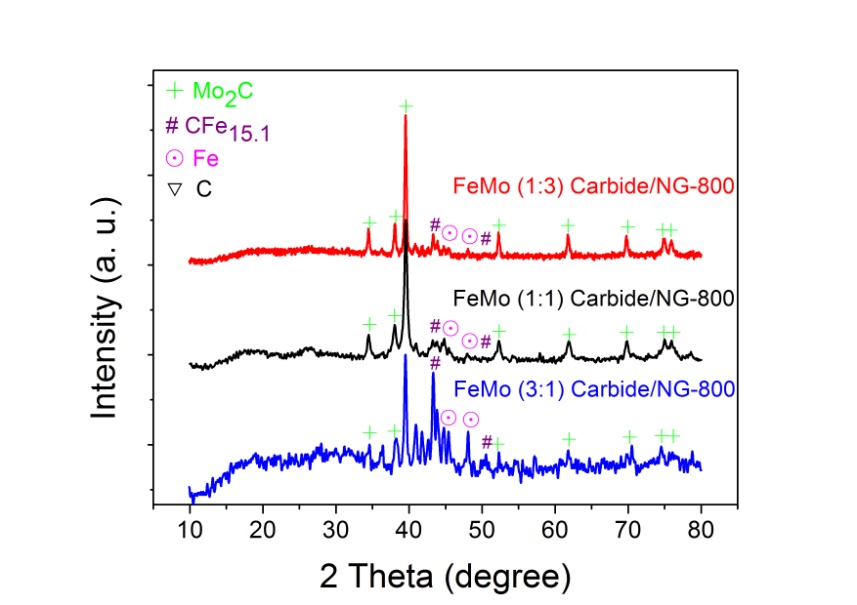


**Fig. S3** XRD pattern of FeMo Carbide/NG catalyst prepared at various Fe/Mo weight ratio after annealing at the same temperature (800 ^o^C).


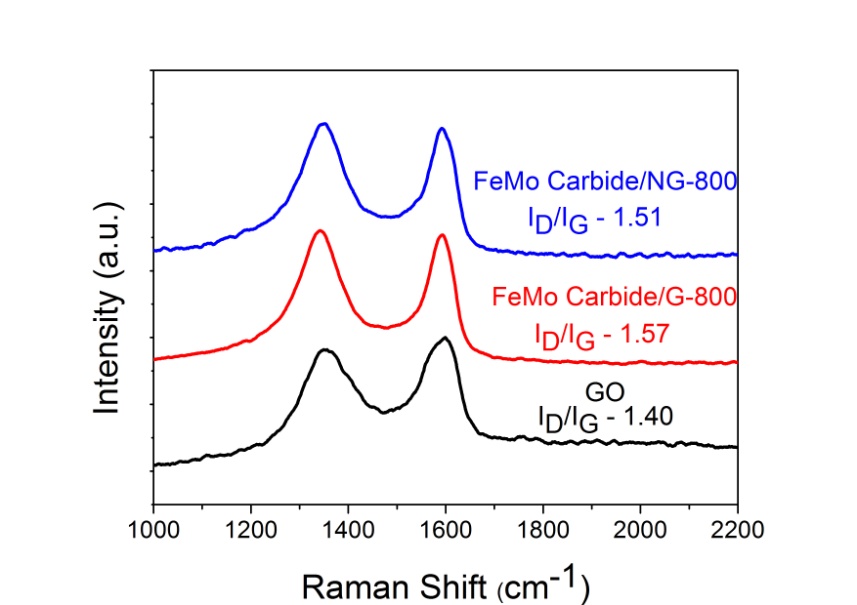


**Fig. S4** Raman spectra of GO, FeMo Carbide/G-800 and FeMo Carbide/NG-800.


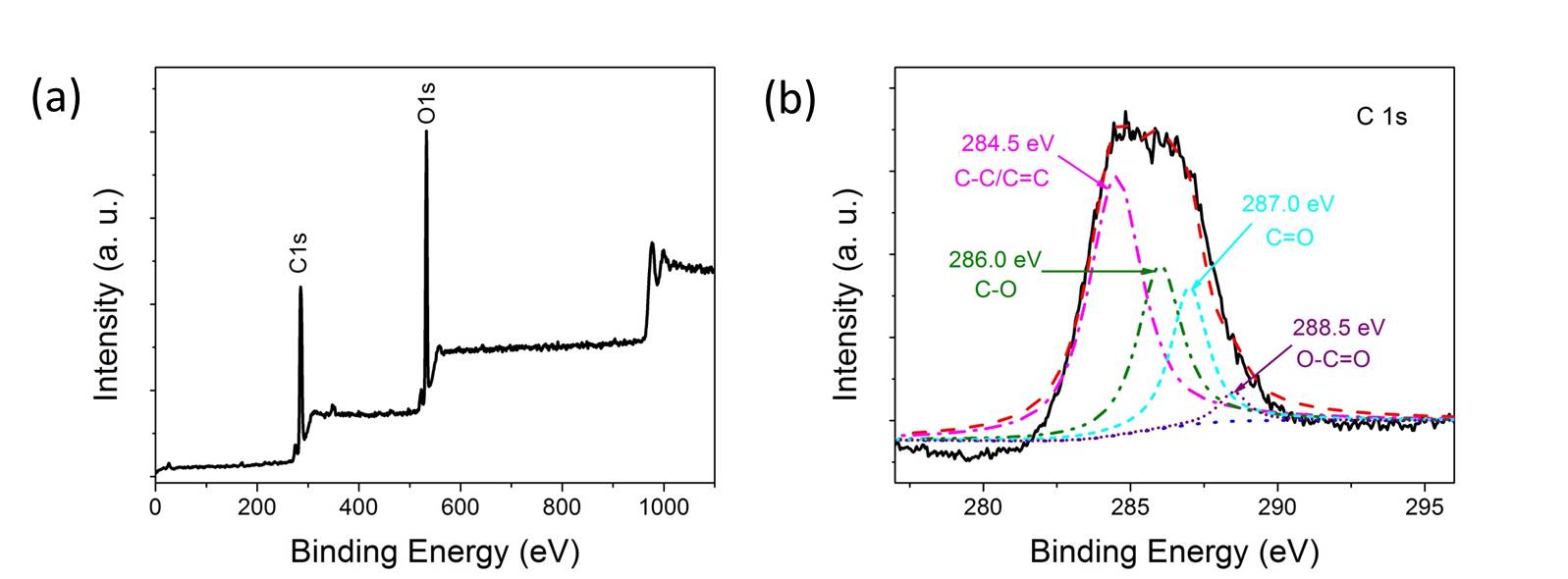


**Fig. S5** High resolution XPS spectra (a) wide scan, (b) C 1s of GO.


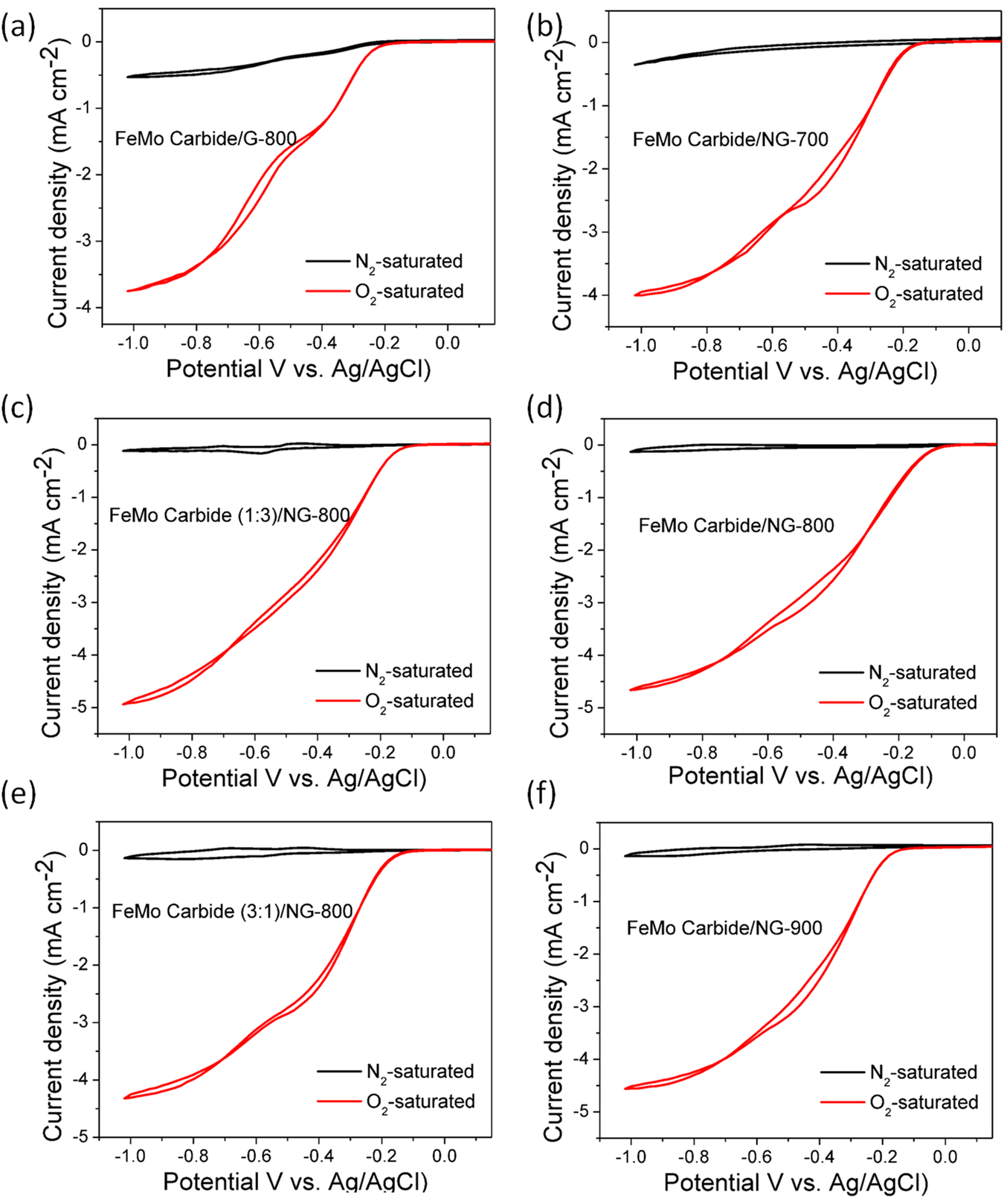


**Fig. S6** Comparative CV curves of (a) FeMo Carbide/G-800, (b) FeMo Carbide/NG-700, (c) FeMo (1:3) Carbide/NG-800, (d) FeMo Carbide/NG-800, (e) FeMo (3:1) Carbide/NG-800, and (f) FeMo Carbide/NG-900, which were loaded on a glassy carbon electrode in O_2_-saturated (read line) or N_2_-purged (black line) 0.1 M KOH solution at a scan rate of 2 mV s^-1^.


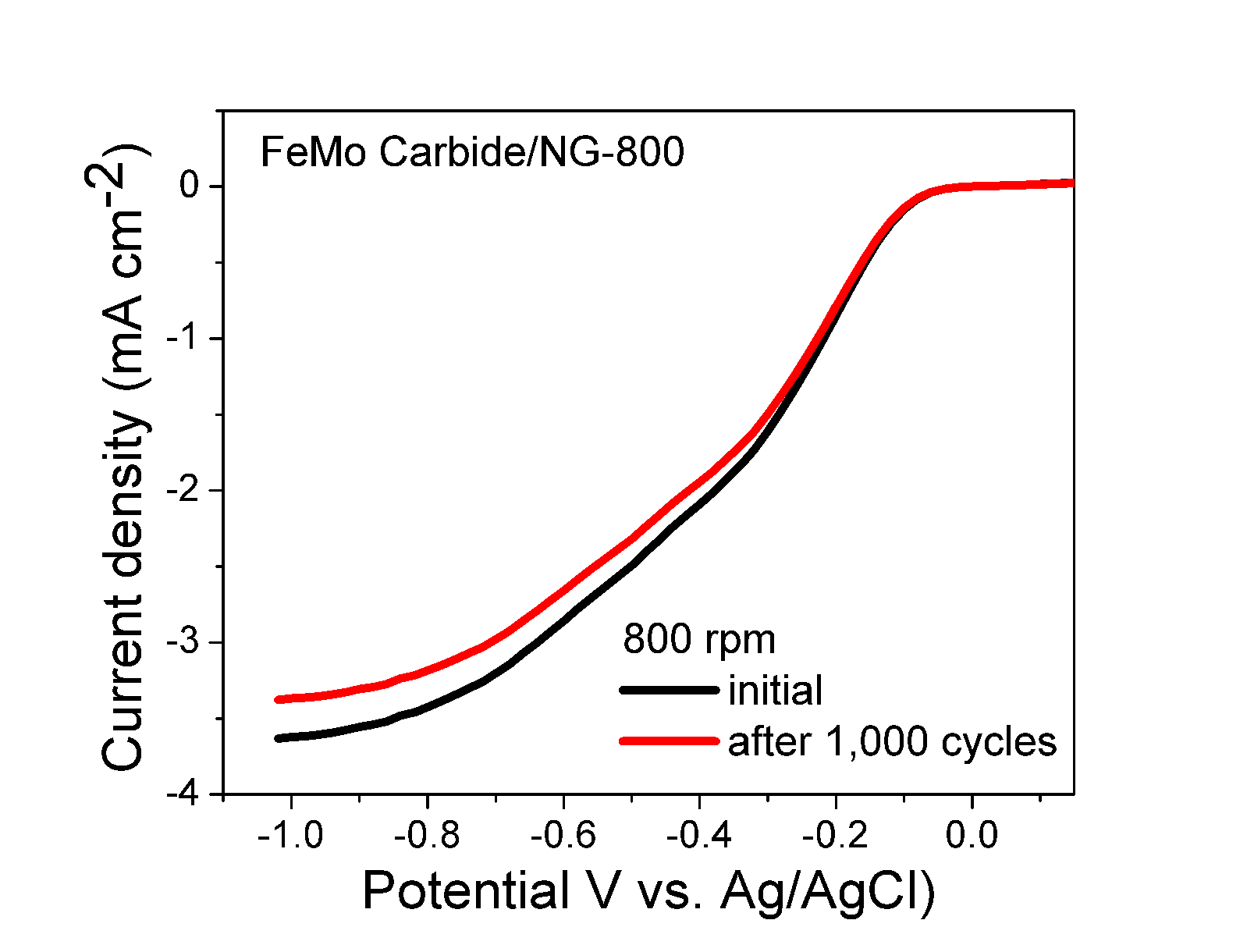


**Fig. S7** RDE curves of the FeMo Carbide/NG-800 before and after 1,000 cycles at a scan rate of 2 mV s^-1^ at the rotation rate of 800 rpm in O_2_-saturated 0.1 M KOH.


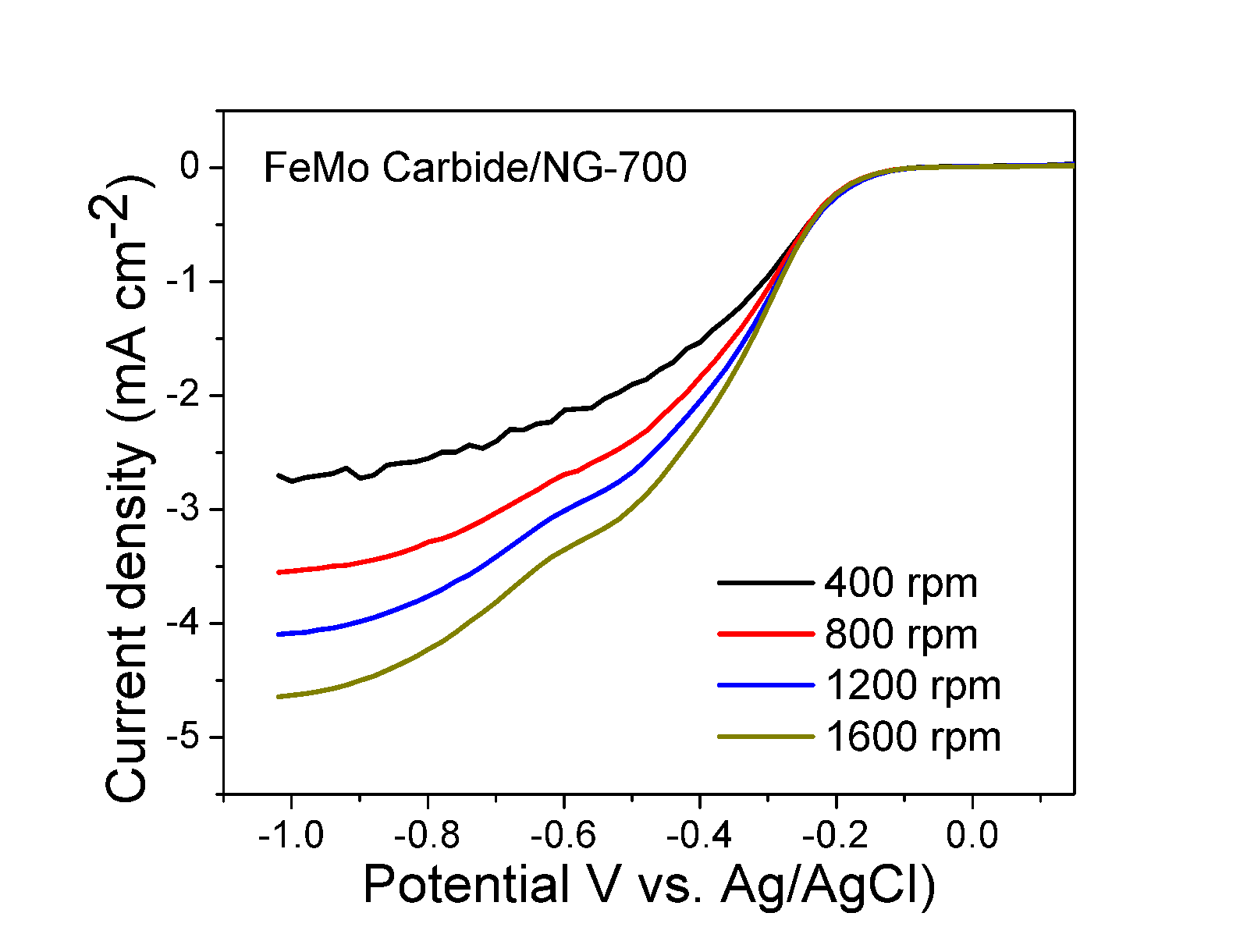


**Fig. S8** RDE curves of the FeMo Carbide/NG-700 after annealing at 700 ^o^C at a scan rate of 2 mV s^-1^ at various rotation rates from 400 to 1600 rpm in O_2_-saturated 0.1 M KOH.


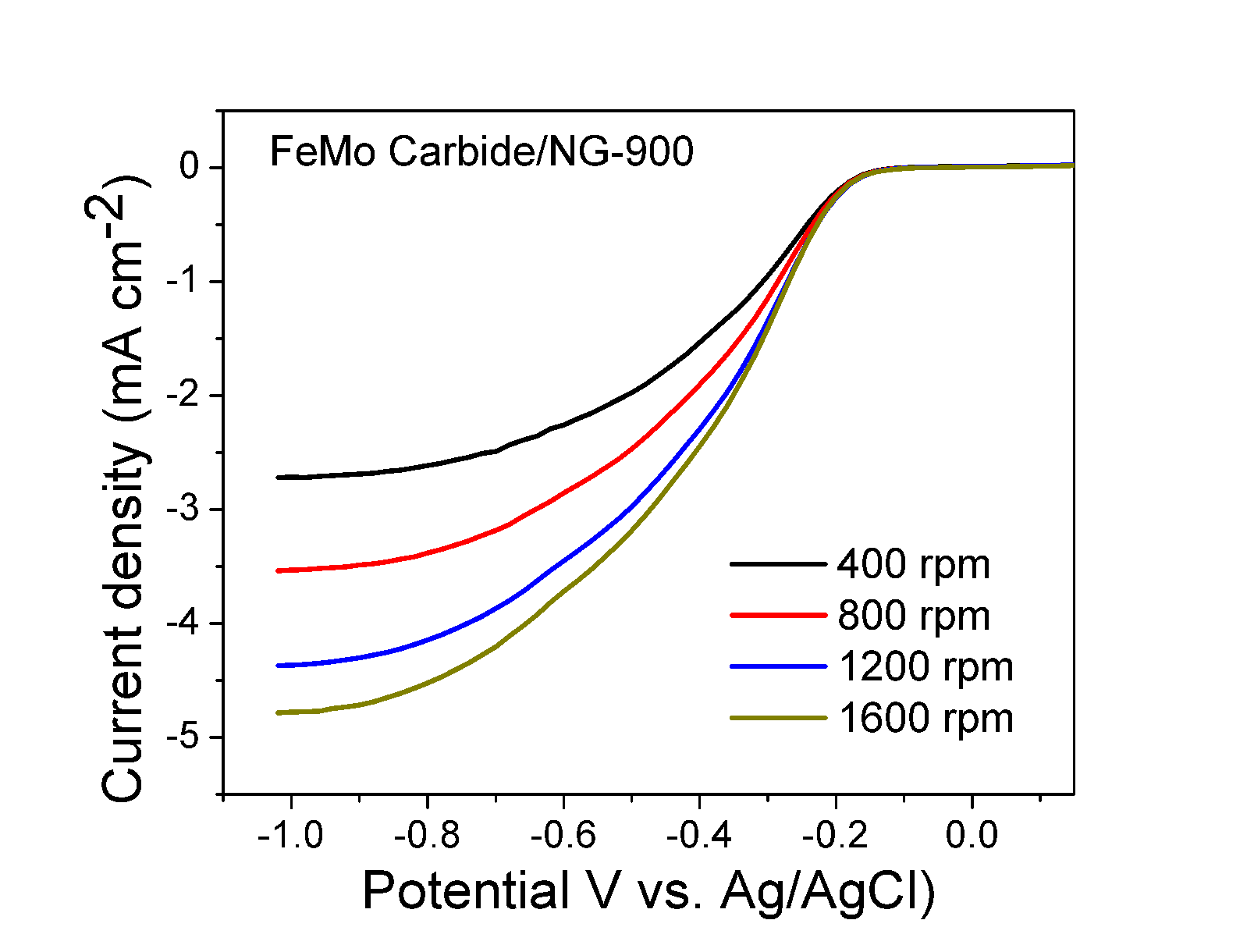


**Fig. S9** RDE curves of the FeMo Carbide/NG-900 after annealing at 900 ^o^C at a scan rate of 2 mV s^-1^ at various rotation rates from 400 to 1600 rpm in O_2_-saturated 0.1 M KOH.


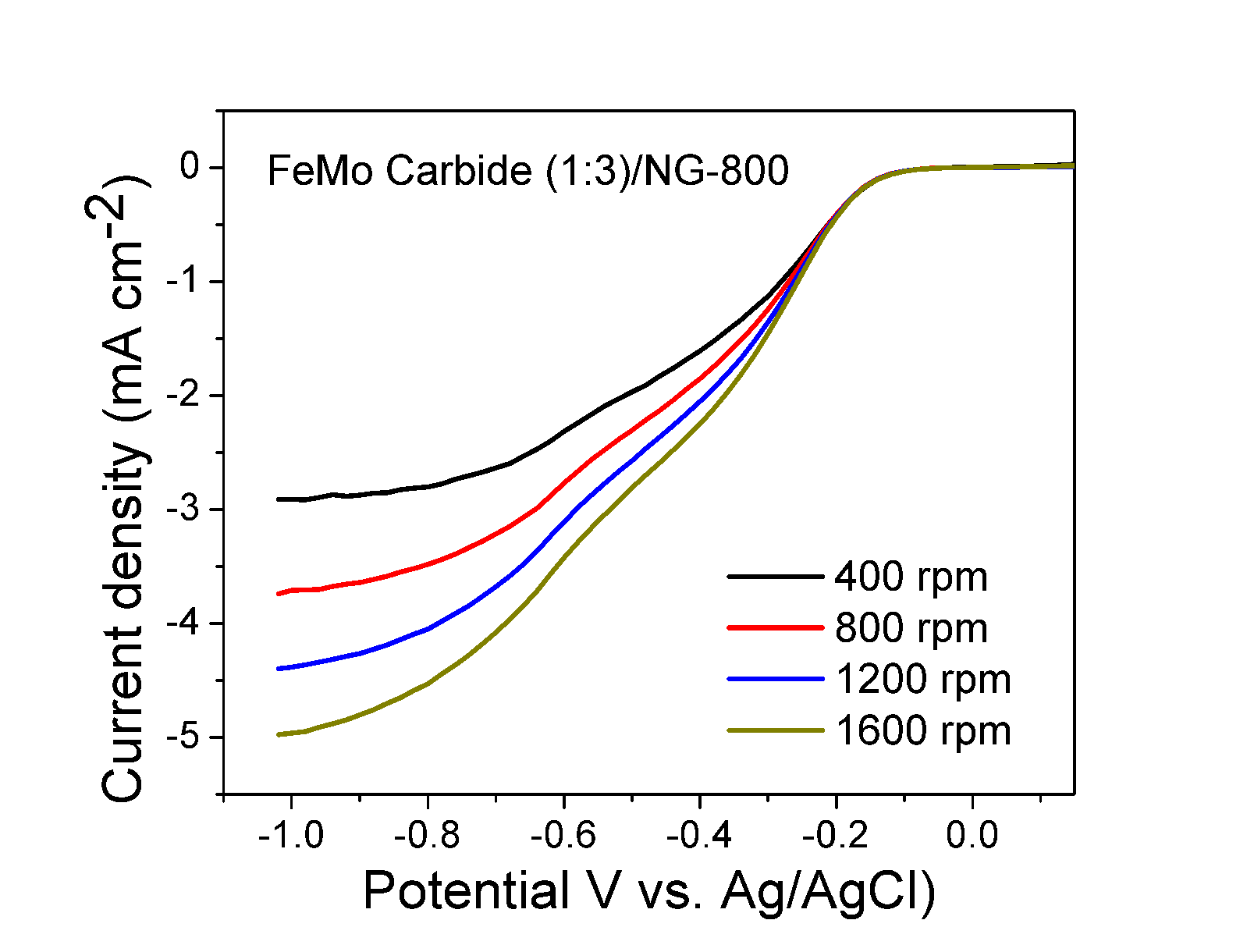


**Fig. S10** RDE curves of the FeMo (1:3) Carbide/NG-800 with Fe/Mo weight ratio (1:3) on the GO surface after annealing at 800 ^o^C at a scan rate of 2 mV s^-1^ at various rotation rates from 400 to 1600 rpm in O_2_-saturated 0.1 M KOH.


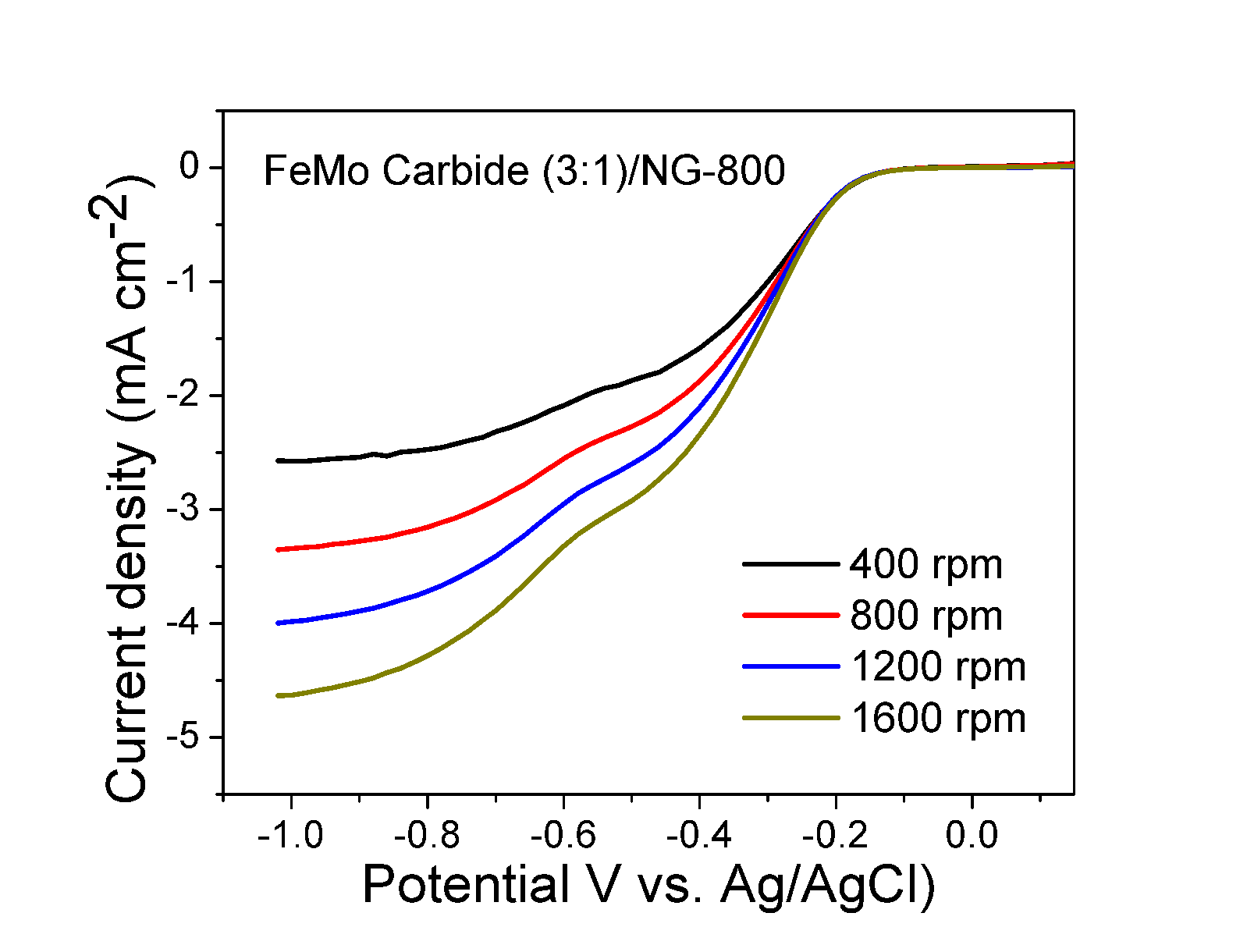


**Fig. S11** RDE curves of the FeMo (3:1) Carbide/NG-800 with Fe/Mo weight ratio (3:1) on the GO surface after annealing at 800 ^o^C at a scan rate of 2 mV s^-1^ at various rotation rates from 400 to 1600 rpm in O_2_-saturated 0.1 M KOH.


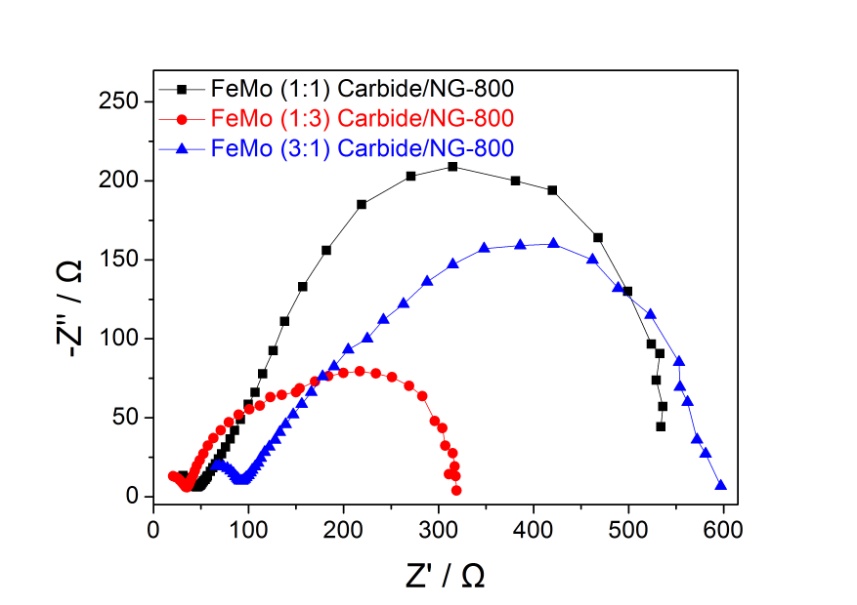


**Fig. S12** Impedance spectra of the FeMo Carbide/NG catalyst prepared at various Fe/Mo weight ratio after annealing at the same temperature (800 ^o^C) measured at 0.73 V.

**Table S1.** Particle size of catalysts as determined by XRD

| Samples | FeMo Carbide/G  -800 | FeMo Carbide/NG  -700 | FeMo Carbide/NG  -800 | FeMo Carbide/NG  -900 | FeMo Carbide (1:3)/NG-800 | FeMo Carbide (3:1)/NG-800 |
| --- | --- | --- | --- | --- | --- | --- |
| Crystal Size (nm) | 24.1 | 19.8 | 21.5 | 22.8 | 23.5 | 23.4 |

**Table S2.** Surface composition of catalysts and distribution of Mo states as determined by XPS

| Samples | Temperature (°C) | Surface composition (%) | | | | | Distribution of Mo states (%) | | |
| --- | --- | --- | --- | --- | --- | --- | --- | --- | --- |
|  |  | C | O | N | Fe | Mo |  | Mo^2+^ | Mo^6+^ |
| GO | - | 68.4 | 31.6 | - | - |  |  | - | - |
| FeMo  Carbide/G  -800 | 800 | 75.9 | 18.4 | - | 1.7 | 4.0 |  | 19.9 | 80.1 |
| FeMo Carbide/NG-800 | 700 | 69.6 | 17.0 | 4.2 | 1.3 | 3.9 |  | 16.3 | 83.7 |
|  | 800 | 75.1 | 15.8 | 4.7 | 1.5 | 3.9 |  | 19.5 | 80.5 |
|  | 900 | 74.3 | 15.0 | 4.9 | 1.5 | 4.3 |  | 17.3 | 82.7 |

**Table S3.** The distribution of N states as determined by XPS

| N States | FeMo Carbide/NG-700 | | FeMo Carbide/NG-800 | | FeMo Carbide/NG-900 | |
| --- | --- | --- | --- | --- | --- | --- |
|  | Peak Position | % N | Peak Position | % N | Peak Position | % N |
| Pyridinic | 398.0 | 23.2 | 398.1 | 23.4 | 398.1 | 17.5 |
| Pyrrolic | 400.1 | 43,7 | 400.1 | 47.0 | 400.0 | 44.4 |
| Graphitic | 401.4 | 33.1 | 401.3 | 29.6 | 401.3 | 38.1 |

**Table S4.** Comparison of electrochemical characterization results for the catalysts compared with the commercial 20 % Pt/C.

| Catalysts | Onset  Potential (V vs. Ag/AgCl) | Half-wave Potential (V) | Current Density at -0.4 V (mA/cm^2^) | Current Density at -0.6 V (mA/cm^2^) |
| --- | --- | --- | --- | --- |
| FeMo Carbide/G-800 | -0.20 | -0.51 | 1.3 | 2.3 |
| Fe Carbide/NG-800 | -0.19 | -0.41 | 1.0 | 1.9 |
| Mo Carbide/NG-800 | -0.17 | -0.39 | 1.3 | 2.1 |
| FeMo Carbide/NG-700 | -0.14 | -0.37 | 2.0 | 2.8 |
| FeMo Carbide/NG-800 | -0.09 | -0.33 | 2.3 | 3.0 |
| FeMo Carbide/NG-900 | -0.12 | -0.36 | 2.2 | 3.1 |
| FeMo (1:3) Carbide/NG-800 | -0.11 | -0.37 | 2.1 | 3.1 |
| FeMo (3:1) Carbide/NG-800 | -0.14 | -0.37 | 1.9 | 2.7 |
| 20 % Pt/C | 0.06 | -0.08 | 3.4 | 3.6 |
